# Molecular weight dependent vertical composition profiles of PCDTBT:PC_71_BM blends for organic photovoltaics

**DOI:** 10.1038/srep05286

**Published:** 2014-06-13

**Authors:** James W. Kingsley, Pier Paolo Marchisio, Hunan Yi, Ahmed Iraqi, Christy J. Kinane, Sean Langridge, Richard L. Thompson, Ashley J. Cadby, Andrew J. Pearson, David G. Lidzey, Richard A. L. Jones, Andrew J. Parnell

**Affiliations:** 1 Ossila Ltd, Kroto Innovation Centre, Broad Lane, Sheffield, S3 7HQ, UK; 2 Department of Chemistry, The University of Sheffield, Sheffield, S3 7HF, UK; 3 ISIS Pulsed Neutron and Muon Source, Science and Technology Facilities Council, Rutherford Appleton Laboratory, Harwell Science and Innovation Campus, Didcot OX11 0QX, UK; 4 Department of Chemistry, Durham, DH1 3LE, UK; 5 Department of Physics and Astronomy, The University of Sheffield, Hicks Building, Hounsfield Road, Sheffield, S3 7RH, UK; 6 Current address: Cavendish Laboratory, University of Cambridge, JJ Thomson Avenue, Cambridge CB3 0HE, UK

## Abstract

We have used Soxhlet solvent purification to fractionate a broad molecular weight distribution of the polycarbazole polymer PCDTBT into three lower polydispersity molecular weight fractions. Organic photovoltaic devices were made using a blend of the fullerene acceptor PC_71_BM with the molecular weight fractions. An average power conversion efficiency of 5.89% (peak efficiency of 6.15%) was measured for PCDTBT blend devices with a number average molecular weight of M_n_ = 25.5 kDa. There was significant variation between the molecular weight fractions with low (M_n_ = 15.0 kDa) and high (M_n_ = 34.9 kDa) fractions producing devices with average efficiencies of 5.02% and 3.70% respectively. Neutron reflectivity measurements on these polymer:PC_71_BM blend layers showed that larger molecular weights leads to an increase in the polymer enrichment layer thickness at the anode interface, this improves efficiency up to a limiting point where the polymer solubility causes a reduction of the PCDTBT concentration in the active layer.

The copolymer PCDTBT (poly[N-9′-heptadecanyl-2,7-carbazole-alt-5,5-(4′,7′-di-2-thienyl-2′,1′,3′-benzothiadiazole)])^1^ is a promising electron donating semiconductor for application in Organic Photovoltaic (OPV) devices. When blended with the fullerene derivative PC_71_BM in a bulk-heterojunction (BHJ) architecture, power conversion efficiencies (PCEs) above 7% have been achieved^2^ with open-circuit voltages V_OC_ in the range of 0.9 V. Although these champion efficiency metrics have since been surpassed by replacing PCDTBT with alternative low bandgap semiconductors^3^, it remains however an attractive polymer for practical applications due to its promising thermal stability, ease of processability and reduced susceptibility to oxidation due to a deep highest occupied molecular orbit (HOMO) level^4^ when compared to polymers like PTB7, which suffer rapid photo-oxidative damage^5^. Indeed, under controlled laboratory conditions, solar cell lifetimes approaching seven years have been estimated for PCDTBT:PC_71_BM OPV devices; a value double that of P3HT:PCBM, making PCDTBT a favourable material for the scale-up of single or tandem-junction OPV devices^4^,^6^,^7^. Recently, PCDTBT has begun to replace P3HT as the benchmark material for OPV devices^8^ and has been used to evaluate new techniques for large-area thin-film deposition^9^,^10^ novel electron donor materials^11^,^12^ and electrode interfacial layers in functional devices^2^,^13^,^14^.

The essentially amorphous nature of as-cast PCDTBT^15^,^16^, its low HOMO level (5.5 eV) and its efficient intermixing with PC_71_BM^16^,^17^ means that it is representative of many new conjugated polymer materials that have been developed. It can be argued therefore that, when compared to the P3HT:PC_71_BM blend system, studies of morphology and composition in PCDTBT:PC_71_BM blends have a broader implication for organic photovoltaic development. However due to a characteristically low degree of crystallinity and long-range phase separation, the nano-structure of PCDTBT:PC_71_BM thin films is difficult to determine unambiguously. This limits the degree to which scanning-probe and electron-microscopy techniques can be applied.

Here, we study vertical stratification in a PCDTBT:PC_71_BM BHJ blend thin film as a function of the molecular weight of the copolymer. This is correlated to the performance of PCDTBT:PC_71_BM OPV devices. Exploring this relationship should help minimise the parameter space when performing a screening process of new material blends for efficient OPV devices, as it provides insight to the interfacial composition of the blend film (i.e. those regions in proximity to the electrodes of the solar cell), such regions have a significant influence on the efficiency of charge extraction. The principal technique that we use to explore vertical stratification is neutron reflectivity. Previous applications of this technique to the characterisation of PCDTBT:PC_71_BM blend thin-films have identified the existence of a PCDTBT enrichment layer close to the PEDOT:PSS interface layer^10^,^15^, providing valuable insight into the degree of film stratification and its influence on device operation.

The molecular weight of donor polymers has been shown to affect the performance of OPVs, particularly for P3HT:PCBM^18^,^19^ blends. Ballatyne et al.^18^ examined the effect of molecular weight on P3HT:PCBM blends and measured the electron and hole mobility in pristine P3HT layers and P3HT:PCBM blends. The hole mobility was relatively constant between 13 kDa–18 kDa and then dropped by an order of magnitude as the molecular weight increased. The measured drop in hole mobility was able to account for the drop in fill factor seen in the blend devices, and it was concluded that the optimum P3HT molecular weight was between 13 kDa–34 kDa. Other work by Ma et al.^19^ showed that the choice of optimum annealing temperature for P3HT:PCBM blends also depended on the molecular weight of the P3HT, with the optimum blend comprising a mixture of high and low molecular weight fractions that formed an interconnected matrix. Muller et al.^20^ showed that F8TBT based OPV devices show a strong correlation of extracted photocurrent with molecular weight until a plateau was reached at a number averaged molecular weight of 10 kDa. Intemann et al.^21^ studied the effect of molecular weight on a ladder-type indacenodithiophene based polymer. The absorptivity and hole mobility of this polymer increased with each 15 kDa increase in molecular weight, effects correlated with an overall improvement in the PCE of fabricated solar cells.

All of these studies indicate the importance of molecular weight on the optimization of OPV efficiency. In PCDTBT:PC_71_BM blends however, relatively little is known regarding the interplay between polymer molecular weight, morphology and device performance. Wakim et al investigated the effect of molecular weight on PCDTBT:PC_60_BM OPV device efficiency^22^. There, four different molecular weight fractions of PCDTBT were evaluated for solar cell performance when blended with PC_60_BM at a ratio of 1:2 wt%. For a M_w_ range of between 37 kDa and 64 kDa fabricated OPV devices yielded PCEs of ~4%. Devices utilising a relatively low M_w_ batch of PCDTBT (22 kDa) however achieved a lower PCE of approximately 2.2%.

In this work we use neutron reflectivity to correlate the relationship between molecular weight and vertical composition of PCDTBT:PC_71_BM films in a 1:4 wt% blend ratio cast onto a layer of PEDOT:PSS^13^,^22^. We show that the molecular weight of the PCDTBT affects the vertical composition, with the use of higher molecular weights resulting in increased PCDTBT enrichment at the interface with PEDOT:PSS. Such vertical composition profiles are beneficial for OPV device performance. Nevertheless for the highest M_w_ batch investigated, reduced solubility of PCDTBT in the casting solvent leads to a reduction in the concentration of the polymer in the blend film bulk, leading to a relative decrease in device efficiency.

## Results and discussion

**Figure 1:**
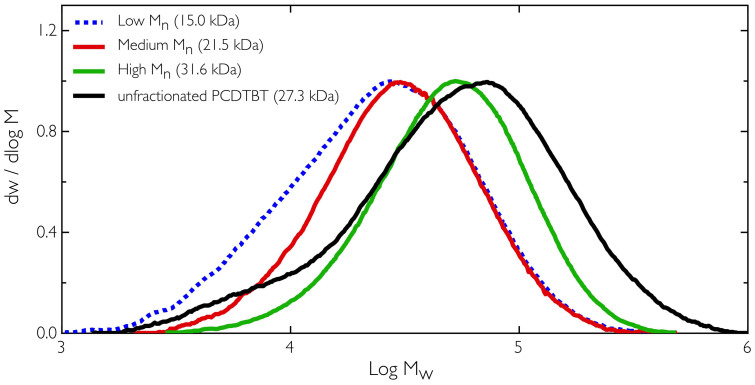
Gel Permeation Chromatography (GPC) data for the three solvent Soxhlet fractions of PCDTBT and the unfractionated PCDTBT.

In Table 1 and Figure 1 the molecular weight distributions and key parameters (M_w_, M_n_, and dispersity) for the different polymer fractions separated by Soxhlet extraction using the solvents chloroform (CH), chlorobenzene (CB) and dichlorobenzene (DCB) are presented. As expected, the process of fractionation reduces the polydispersity and lowers the M_w_ relative to the unfractionated material. This resulted in a significant variation in M_n_ with values of 15 kDa, 21.5 kDa and 34.9 kDa respectively for the CH, CB and DCB Soxhlet fractions respectively, and provided a relatively wide range over which to test the effect of molecular weight on thin film OPV devices. By comparing device data with the vertical composition data we demonstrate that increased molecular weight is correlated with improved power conversion efficiency up to the limit of PCDTBT solubility.

The various Soxhlet fractions were dissolved in a common solvent for the fabrication of organic photovoltaic devices. Here, CB was selected due to its intermediate boiling point (132°C) and good film forming properties. However, for high molecular weights, the solubility of the PCDTBT becomes a limiting factor. While the use of DCB would allow more of the material to be solubilised, we have previously shown that this is a relatively small improvement over the material solubility in CB^23^. We have also found that the use of DCB leads to poor OPV device performance, possibly due to residual solvent effects, PCBM aggregation and crystallite formation.

**Figure 2:**
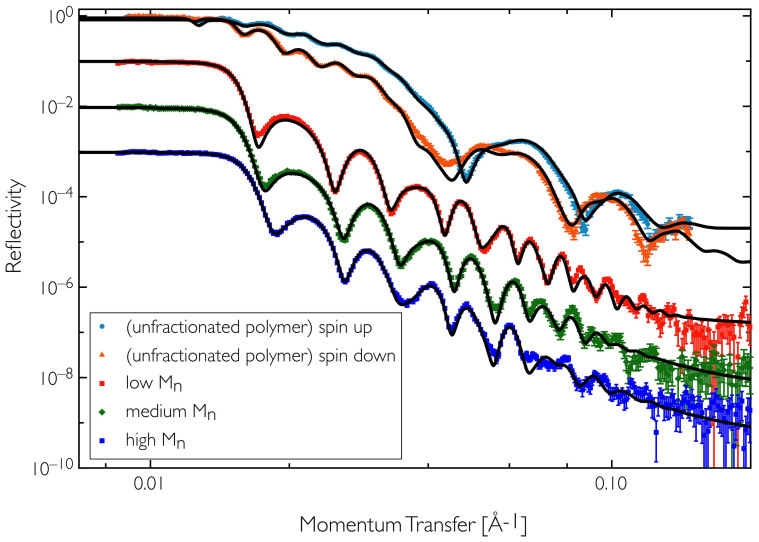
Specular neutron reflectivity data for the different Soxhlet fractions of PCDTBT polymer studied and the unfractionated polymer, showing data with associated errors and fits to the data (black lines). The data are offset by factors of 10 for clarity. The unfractionated data was measured using a magnetic reference layer (NiFe) with polarised neutrons and so we have measured spin up and spin down to give two reflectivity datasets. The scattering length density of pure PCDTBT was taken as 1.34 × 10^−6^Å^−2^ (from previous NR thin film measurements of PCDTBT) and PC_71_BM as 4.46 × 10^−6^ Å^−2^ was calculated using the NIST online database.

**Figure 3:**
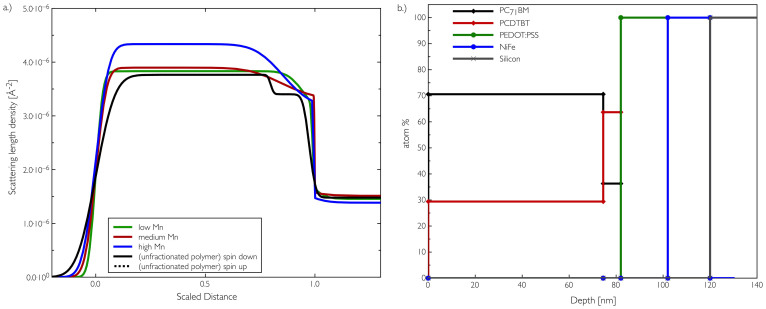
a.) Profiles generated from the NR fitted data showing the PCDTBT PCBM vertical profile on PEDOT:PSS. The distance is scaled to the onset of the PEDOT:PSS layer to aid comparisons in the profiles. b.) Vertical depth profile measured for the unfractionated polymer as measured via ion beam analysis.

Neutron reflectometry measurements were carried out at the ISIS spallation neutron source (Oxfordshire, UK) using the instrument CRISP^24^,^25^. The measured reflectivity data for the various blend films are shown in Figure 2 with the key data from the profiles shown in Figure 3 and summarised in Table 2. The model fitting gives the scattering length density (SLD) profile normal to the film surface, which allows us to deduce the vertical composition profiles, as shown in Figure 3. The different layers (silicon, PEDOT:PSS and blend) are readily identifiable due to their different scattering length densities, allowing us to determine changes in composition within the blend layer itself.

Using the calculated scattering length density for pure PCDTBT and pure PC_71_BM, we can estimate the percentage of PCDTBT in the bulk part of the thin films as 19% and 17% for the medium (CB) and low (CH) M_w_ fractions respectively and just 3% for the high M_w_ fraction. In order to validate the vertical structuring in this system we have also performed ion beam analysis on the unfractionated sample where NR experiments had provided two contrasts due to the presence of NiFe. Although this technique has somewhat lower resolution than NR, it does however qualitatively agree with the vertical profile measured using neutron reflectivity. The three datasets in SI Figure 1 were fitted simultaneously to generate the profile in Figure 3b.

**Figure 4:**
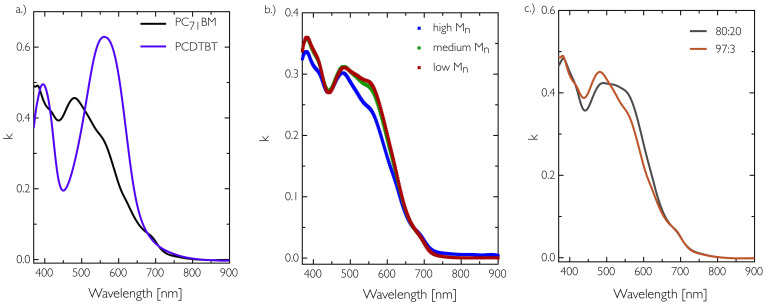
Extinction coefficient data of the thin film OPV blend and pure constituent samples. a.) Pure PCDTBT and PC_71_BM thin films. b.) Data for the low, medium and high M_n_ OPV blend layers. c.) A linear combination mixing model based on the neutron reflectivity derived composition for the OPV films.

To further confirm the composition of the OPV blends in the active layer, the ellipsometry data in the optically absorbing region was modelled to extract the extinction coefficient. The data in Figure 4a shows the measured extinction coefficient for pure thin films of PC_71_BM and PCDTBT and matches well with previous studies^22^,^23^,^26^. The OPV blend data for the fractionated material is displayed in Figure 4b and clearly shows a difference in their optical absorption, the high M_n_ blend having a reduction above 500 nm due to a reduction in the content of PCDTBT, consistent with the NR data. In Figure 4c a linear combination mixing model based on the contributions of the two components was made, clarifying that the neutron reflectivity derived blend compositions in the bulk of the active layer are accurate.

Of particular interest though is the formation of an enrichment layer of PCDTBT at the interface to the PEDOT:PSS that varies in size with the different molecular weight fractions. This interfacial region could be expected to facilitate efficient hole extraction to the anode in functional devices. The width of this layer increases from 12 nm to 31 nm from the CH (low) to the medium (CB) molecular weight fractions but then decreases slightly to 25 nm for the high (DCB) fraction due to the lower overall PCDTBT content (46% less PCDTBT). Our understanding of this PCDTBT polymer enriched layer comes from the preferential wetting and layering of PCDTBT on the PEDOT:PSS substrate surface^15^. The final spin coated thin film morphology depends on the solubility of the materials and surface energies of the components of the blend, and importantly the substrate surface energy. The vertical segregation is also determined by the liquid-vapour surface tension differences. Although the dominant factor determining vertical composition will be the thermodynamics of the blend mixture^27^, for the case of the high molecular weight blend, the composition is dramatically different to the other molecular weight films and so will alter the strength of this segregation to the anode interface.

There has been a large body of work looking at layering and stratification in polymer polymer blend systems during the evolution of spin coated thin films^28^,^29^. Understanding this stratification or layering in blends of OPV relevant materials has helped to understand some of the effects of processing and the relative improvements in device efficiency^30^,^31^,^32^. Indeed OPV applicable simulations of these enriched and layered structures are now possible to model and simulate using Cahn-Hilliard theory^33^.

**Figure 5:**
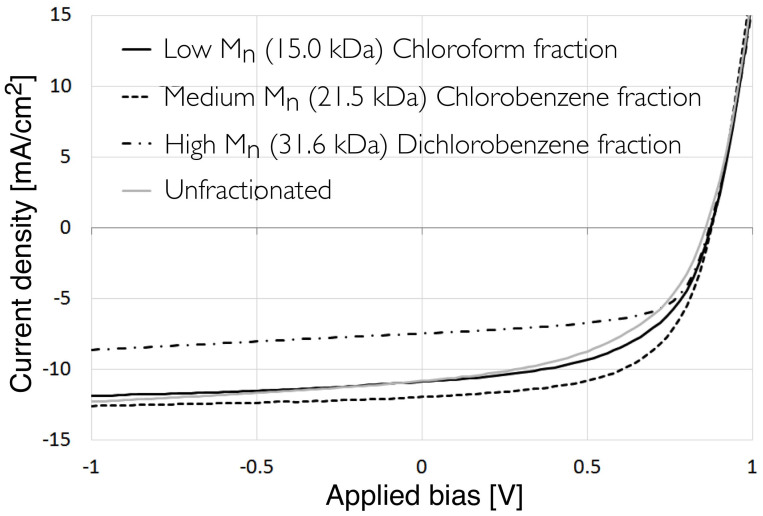
Representative JV curves for OPV devices fabricated from the different molecular weight fractions as well as the unfractionated PCDTBT.

To evaluate the effect of these PCDTBT enrichment layers and changes in the bulk film composition on device performance, OPV devices were fabricated using identical blend solutions (see experimental section for details of device architecture). The results of which are presented in Table 3 and the Supplementary section, with the JV characteristics for the best devices from each molecular weight fraction shown in Figure 5. Interestingly the enrichment layers seem to be a general property of this blend ratio as they are observed when the layers are spin coated on Molybdenum Oxide (see SI Figure 2), however they are not observed when spun at much higher spin speeds of 1500 rpm and 3000 rpm (see SI Figure 3). We think this difference is due to the drying dynamics for the different spin speeds, higher spin speeds do not allow the blend time to fully reach the layered evolution of the wetting and layered states seen in these slower spin speed prepared samples^34^.

The results of this device study demonstrate the dramatic effect of polymer molecular weight on device performance, with a 60% relative difference in average device efficiency between the best and worst performing devices. By performing a Soxhlet extraction using CH, even the lowest M_n_ fraction had markedly improved performance compared to the unfractionated sample, improving in average PCE from 4.3% to 5.02%. It is known that fractionation removes oligomeric residue from the unfractionated polymers and results in an improvement in the fill factor of OPV devices; an effect observed in other OPV systems^35^. Here, device PCE is maximised for the medium M_n_ (CB) fraction, having an average PCE of 5.89%; a value that then drops rapidly for the highest M_n_ fraction that has a PCE of 3.70%.

The origin of these differences can be partly explained by the blend ratio of the polymer to the fullerene, with both the unfractionated and DCB fraction having significantly lower polymer content. To investigate this, devices were fabricated using the unfractionated material in which the blend ratio between PCDTBT and PC_71_BM was varied between 1:2 and 1:4 wt%. Here it was found that a blend ratio of 1:2 gave a maximum PCE of 5.50%; an efficiency similar to that achieved using the CB fraction. Note however that in this case the solution preparation process (stirring in CB for 24 hours at elevated temperature) is effectively performing a Soxhlet extraction process and so is not detailed further.

Overall, the lower solubility, higher surface roughness (as determined by NR) and blend ratio of both the crude and DCB fractions indicate that these are likely to be the major causes of poor device performance and reduced consistency in device efficiency. Devices fabricated from the higher M_n_ DCB fraction were characterised by a reduction in the short circuit current density (J_sc_) of 33% (7.78 mA cm^−2^ compared to 11.64 mA cm^−2^ for the best medium M_n_ fraction). We correlate this reduction to a drop in optical absorption efficiency of the blend films at wavelengths longer that 525 nm, as the amount of PCDTBT in these films is reduced. Optical reflectance measurements on the OPV devices based on the higher M_n_ DCB fraction show differences in the absorption spectra, with a relatively higher reflectivity beyond 525 nm resulting from a reduction in relative PCDTBT concentration (see SI Figure 4)^36^. This agrees with the ellipsometry derived extinction coefficient measurements on the NR samples. For the crude fraction it was also noted that there were some signs of erratic JV sweep behaviour, a feature typical of trapping and de-trapping caused by impurities, indicating as expected that the Soxhlet process helps to improve the purity of the polymer.

We find that the CH and CB fractions not only produce higher efficiency OPV devices but the devices have better pixel-to-pixel uniformity. However, although there are no significant variations in blend ratio, film thickness, optical absorption or energy levels between these polymer fractions there is still a statistically significant difference in device efficiency (5.02% and 5.89% average PCE for CH and CB fractions respectively). Here, the reduction in efficiency mainly results from a reduction in fill factor of the CH-extracted polymer. From Figure 5 however, we note that at a reverse bias of −1 V, the short-circuit currents are similar (11.9 and 12.6 mA/cm2 for CH and CB fractions respectively). We suspect therefore that the major difference in performance between the CH and CB fractions is caused by variations in hole extraction efficiency rather than charge generation efficiency.

To test this hypothesis, hole mobility measurements were made on the different PCDTBT M_w_ fractions using organic field effect transistors (OFETs). OFETs were created using a top-gate bottom-contact architecture using a large channel width (30 mm) and length (50 to 150 μm) to minimise contact effects. In these devices PMMA was used as the gate insulator and high conductivity PEDOT:PSS was used as the gate. By varying the channel length, we found that the devices were channel dominated (i.e. Poole-Frenkel) rather than contact limited; a result that gives us a high degree of confidence that there is a statistically significant difference between the materials. Interestingly though, we found that the lower molecular weight fraction had the higher hole-mobility. However, the absolute difference in mobility is relatively low with around 30% variation (average mobility for low and medium M_n_ fractions being 4.33 × 10^−4^ cm^2^/Vs and 3.3 × 10^−4^ cm^2^/Vs respectively, as seen in SI Figure 5). As such, the differences in mobility may be as much due to the film forming and aggregation properties of the materials as the molecular weight. While it is known that the addition of PCBM can significantly increase the solubility of the PCDTBT^23^ and that the mobility of a blend film is different to that of a pure polymer film, the vertical phase composition and enrichment layers at the surface clearly complicate the analysis of such blend films. However, since the higher molecular weight polymer films have a slightly lower mobility, we ascribe the improved charge extraction to the presence of an enrichment layer of PCDTBT at the surface of the PEDOT:PSS hole extraction interface.

In conclusion, we have compared the characteristics of OPV devices using one of four different PCDTBT polymer samples with different molecular weights, when mixed with PC_71_BM in a 1:4 wt% blend ratio. We find that a polymer having a number average molecular weight of 21.5 kDa produces devices having an average PCE of 5.89% (6.15% maximum). This value is a ~40% higher than efficiencies from the unfractionated polymer. Our neutron reflectivity measurements show that the medium number average molecular weight based devices have the thickest polymer enrichment layer along with a bulk layer composition that is similar to that of the blend solution before deposition.

## Methods

PCDTBT was synthesized as reported by Yi et al.^23^ with the product purified using Soxhlet extraction. The material was washed for 24 hours in each of methanol, acetone and cyclohexane to remove any monomers, end capping components and other impurities to provide the unfractionated polymer. Separation of the different molecular weight fractions was performed by Soxhlet extraction of the polymer consecutively for 24 hours in chloroform, chlorobenzene, which produced materials of increasing molecular weight. After Soxhlet of the fraction using chlorobenzene the remaining material in the cellulose thimble was made up in a flask of Dichlorobenzene and heated to 120°C for an hour to allow the remaining PCDTBT polymer to dissolve. This solution was subsequently filtered using a 0.2 μm Fluoropore PTFE filter membrane (Sigma-Aldrich). Polymer solutions in 1,2,4-trichlorobenzene at 100°C were used as samples for GPC analysis. The GPC curves were obtained by the RI-detection method, which was calibrated with a series of polystyrene narrow standards (Polymer Laboratories).

OPV devices were prepared using a standardised procedure^23^ with all materials and components supplied by Ossila Limited and used as received unless specified. Pre-patterned ITO was cleaned using Hellmanex III (TM), IPA and hot 10% NaOH before being coated with PEDOT:PSS (Clevios AI 4083) by spin coating at 5000 rpm for 30 s under ambient conditions to produce an approximate film thickness of 40 nm. After coating, the PEDOT:PSS coated devices were immediately placed on a hotplate at 150°C until transfer to a glovebox where the rest of the processing was undertaken. The PCDTBT fractions were dissolved in anhydrous chloroform (Sigma Aldrich) using a stirrer bar for 24 hours on a hotplate set to 80°C before being blended with dry PCBM to the desired ratio and stirred at 80°C for another 2 hours. After cooling the solutions were filtered through a 0.45 μm PTFE filter before use. The blend solutions were deposited by spin coating at spin speeds between 600 rpm and 1200 rpm (low M_n_ 700 rpm, medium M_n_ 1250 rpm, high M_n_ 600 rpm and the unfractionated polymer 610 rpm) to produce layers ~75 nm thick as determined by surface profilometry. Note that the higher number average molecular weight fractions did not fully dissolve and so were filtered through a 5 μm PTFE filter prior to blending and generally required lower spin speeds, indicating a reduced blend ratio in agreement with the Neutron reflectivity data. Devices were finally completed by thermally evaporating 2.5 nm Calcium and 100 nm of Aluminium for the cathode and then encapsulated using inert epoxy and a glass coverslip. Thermal annealing was performed at 80°C for 10 minutes on a hotplate in glovebox before encapsulation. Current-voltage sweeps were taken using a Keithley 237 with a calibrated aperture mask under a Newport 92251A-1000 AM1.5 solar simulator calibrated with an NREL certified silicon reference cell.

To provide high quality statistical data from research-based devices with moderate yield, a total of four substrates, each with four pixels were fabricated for each fraction, yielding a total of 16 pixels. To avoid any subjective analysis of yield the data presented here is simply the average of the best 12 out of 16 pixels (top 75%). All the data is plotted for completeness in SI Figure 6.

Optical reflectivity was recorded using a Shamrock ANDOR spectrograph and a collimated Tungsten light source. The OPV device samples were measured at an angle of 15° for all the samples with the data normalised to the reflectivity at 510 nm. This procedure was based on previous studies that have shown this to be constant within the range of PCDTBT:PC_71_BM composition^36^.

Spectroscopic ellipsometry of the samples was measured using a Woolam M2000V ellipsometer for the samples (excluding the sample on NiFe), as this allowed the total layer thickness to be measured by fitting a Cauchy model to the optically transparent region (800 nm–1000 nm), this data along with fits in the optically transparent region are presented in SI Figure 7. The extinction coefficient (k) for the OPV blend layer PCDTBT was extracted by using a B-Spline model incorporating a Kramers-Kronig model, over the absorption range 370 to 800 nm and beyond to 1000 nm.

The neutron reflectivity data were measured on the instrument CRISP at the ISIS spallation neutron source, which is a time of flight instrument using neutrons with incident wavelengths of 0.5 Å to 6.5 Å^34^. Three angles were measured to cover the total wavevector range, with the unfractionated sample including a magnetic reference layer^37^. This procedure produced two reflectivity datasets, one spin up and one spin down. The scattering length density of pure PCDTBT was taken as 1.34 × 10^−6^Å^−2^ (from previous NR thin film measurements of PCDTBT) and PC_71_BM as 4.46 × 10^−6^ Å^−2^ was calculated using the NIST online database. The data was simulated using the total layer thickness from the ellipsometry as a starting point and fitted using the scheme of Névot and Croce, with the two spin-states simultaneously data fitted using MotoFit^38^. The depth profiles without normalising to thickness are shown in SI Figure S8 and scanning probe microscopy images of the sample surface are in SI Figure S9 along with the extracted root mean square surface roughness.

The ion beam depth profile in Figure 3 (b) is the result of a simultaneous fit of three sets of data using the Datafurnace ion beam analysis code^39^. The Rutherford Backscattering (RBS) and elastic back scattering (EBS) mainly confirm the depth of the silicon substrate and NiFe layer, and show the sulphur that is present in some of the organic components. The elastic recoil detection analysis (ERDA) spectrum is the one that shows the variation in hydrogen concentration as a function of depth, which in this particular instance is very useful because of the natural contrast between PC_71_BM (H sparse) and PCDTBT, which is relatively H-rich. The application of ion beam analysis techniques to polymer materials is reviewed in detail elsewhere^40^,^41^.

## Author Contributions

D.L. wrote the grant that made this work possible. The PCDTBT polymer was synthesized and Soxhlet fractionation was performed by H.Y. and A.I. The NR samples and OPV devices were made by J.W.K., whilst the OFET measurements were performed by P.P.M. Parnell, R.A.L.J. and S.L. wrote the proposal for facility access to ISIS. A.J.P., A.J.C. and C.J.K. performed the measurements at the ISIS spallation Neutron Source. A.P. and D.L. came up with the reflectance measurements to confirm the NR data. R.L.T. performed the ion beam analysis, including the measurements and data modelling. All of the authors contributed to the writing of the manuscript.

## Supplementary Material

Supplementary Information Supporting Information

## Figures and Tables

**Table 1:** Molecular weight data for the separate PCDTBT fractions. Polymer solutions in 1,2,4-trichlorobenzene at 100 °C were used as samples for GPC analysis. The GPC curves were obtained by the RI-detection method, which was calibrated with a series of narrow polystyrene standards (Polymer Laboratories)

Polymer Soxhlet Fraction	M_n_ (kDa)	M_w_ (kDa)	Dispersity
Chloroform (CH)	15.0	34.8	2.32
Chlorobenzene (CB)	21.5	38.8	1.8
Dichlorobenzene (DCB)	34.9	61.6	1.76
Unfractionated	27.3	82.2	3.01

**Table 2:** Neutron scattering parameters for the thin films composed of different molecular weight fractions

PCDTBT M_w_	SLD of bulk layer (Å^−2^)	% PC_71_BM in bulk layer	Polymer enriched layer thickness [nm]
Un-fractionated	3.76e-6	79	11
Low	3.84e-6	81	12
Medium	3.90e-6	83	31
High	4.33e-6	97	25

**Table 3:** Key metrics for OPV devices fabricated from different OPV fractions. The average data presented here is simply the average of the best 12 out of 16 pixels (top 75%). The device architecture is ITO/PEDOT:PSS/Active layer PCDTBT:PC_71_BM/Calcium (2.5 nm)/Aluminium (100 nm)

Polymer fraction	Maximum (PCE) [%]	Average PCE [%]	Average V_oc_ [V]	Average J_SC_ [mA/cm^2^]	Average Fill Factor [%]
Unfractionated	4.62	4.30 ± 0.27	0.86 ± 0.01	−10.48 ± 0.30	47.8 ± 1.8
Low	5.10	5.02 ± 0.07	0.87 ± 0.01	−10.78 ± 0.11	53.6 ± 0.7
Medium	6.15	5.89 ± 0.17	0.88 ± 0.01	−11.64 ± 0.55	57.7 ± 2.2
High	4.12	3.70 ± 0.34	0.86 ± 0.01	−7.79 ± 0.39	55.4 ± 6.0
