# Supplementary material for: Molecular weight dependent vertical composition profiles of PCDTBT:PC71BM blends for organic photovoltaics
Source: Sci Rep. 2014 Jun 13;4:5286. doi: 10.1038/srep05286 (PMC4055896; doi:10.1038/srep05286)
Supplement: Supplementary Information — Supporting Information [file srep05286-s1.doc]

Molecular weight dependent vertical composition profiles of PCDTBT:PC_71_BM blends for organic photovoltaics

James W. Kingsley*, Pier Paolo Marchisio*

Hunan Yi¶, Ahmed Iraqi¶

Christy J. Kinane∞, Sean Langridge∞

Richard L. Thompson#

Ashley J. Cadby†, Andrew J. Pearson†, David G. Lidzey†, Richard A. L. Jones†, Andrew J. Parnell†

*Ossila Ltd, Kroto Innovation Centre, Broad Lane, Sheffield, S3 7HQ, UK

¶Department of Chemistry, The University of Sheffield, Sheffield, S3 7HF, UK

∞ISIS Pulsed Neutron and Muon Source, Science and Technology Facilities Council, Rutherford Appleton Laboratory, Harwell Science and Innovation Campus, Didcot OX11 0QX, UK

#Department of Chemistry, Durham University, Durham, England.

†Department of Physics and Astronomy, The University of Sheffield, Hicks Building, Hounsfield Road, Sheffield, S3 7RH, UK


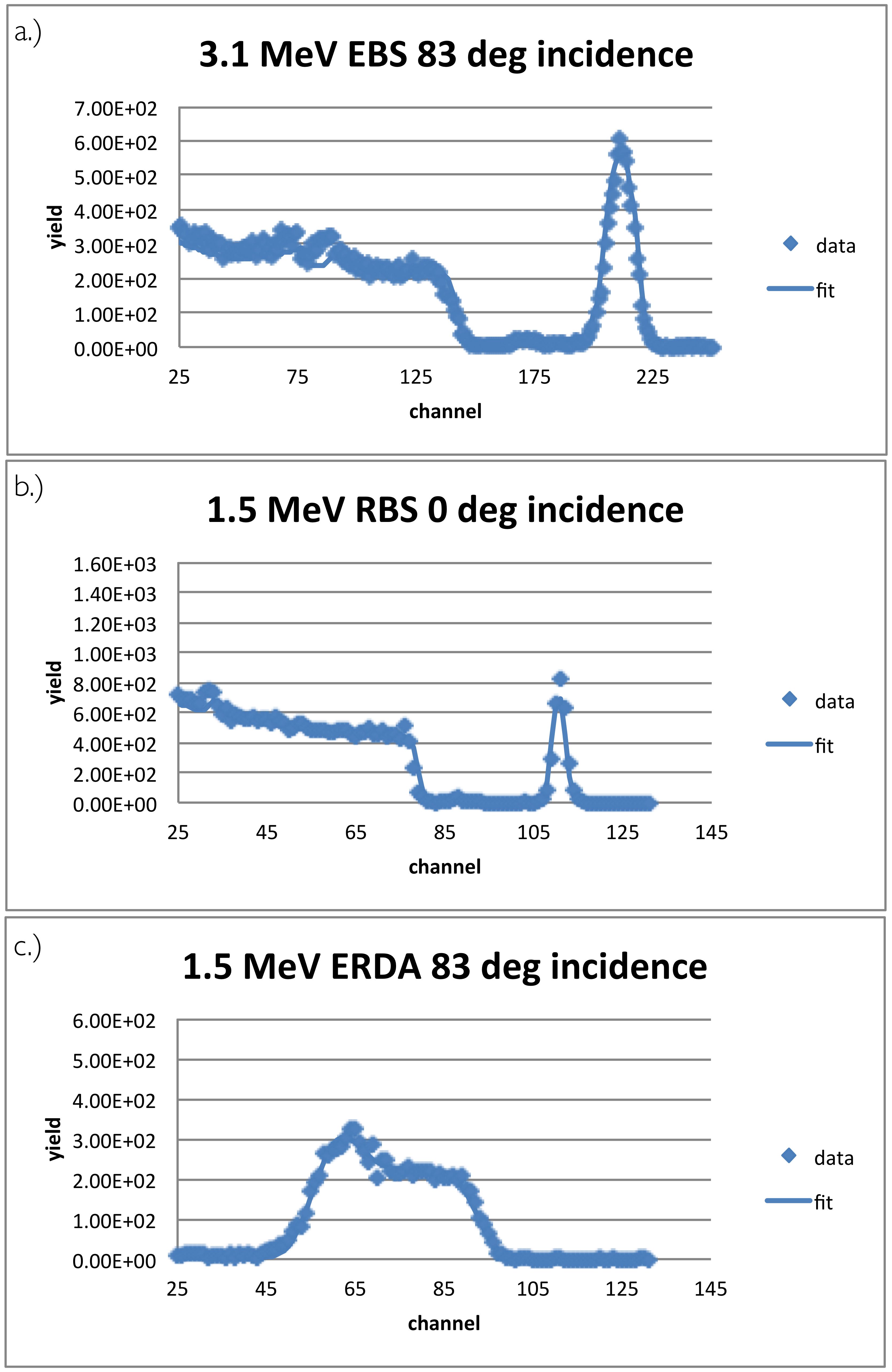


**Figure SI 1.** Ion beam analysis of the (unfractionated) PC71BM:PCDTBT blend film. a.) is the elastic back scattering (EBS), b.) the Rutherford Backscattering (RBS) signal and c.) is the elastic recoil detection analysis (ERDA) spectrum.


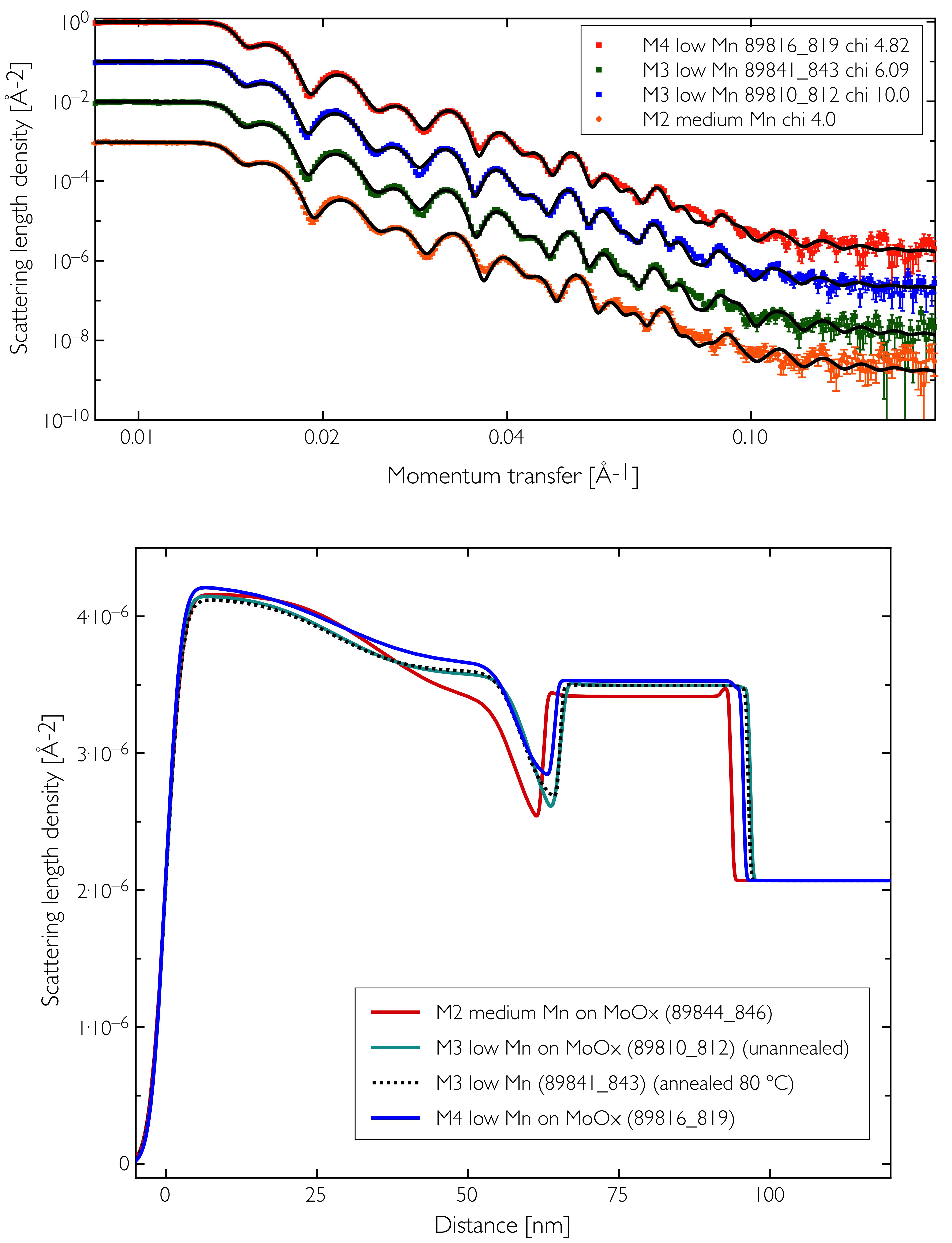


**Figure SI 2.** Neutron reflectivity data for spin coated 1:4 blend layers of PCDTBT:PC_71_BM at spin coated on Molybdenum oxide surfaces.


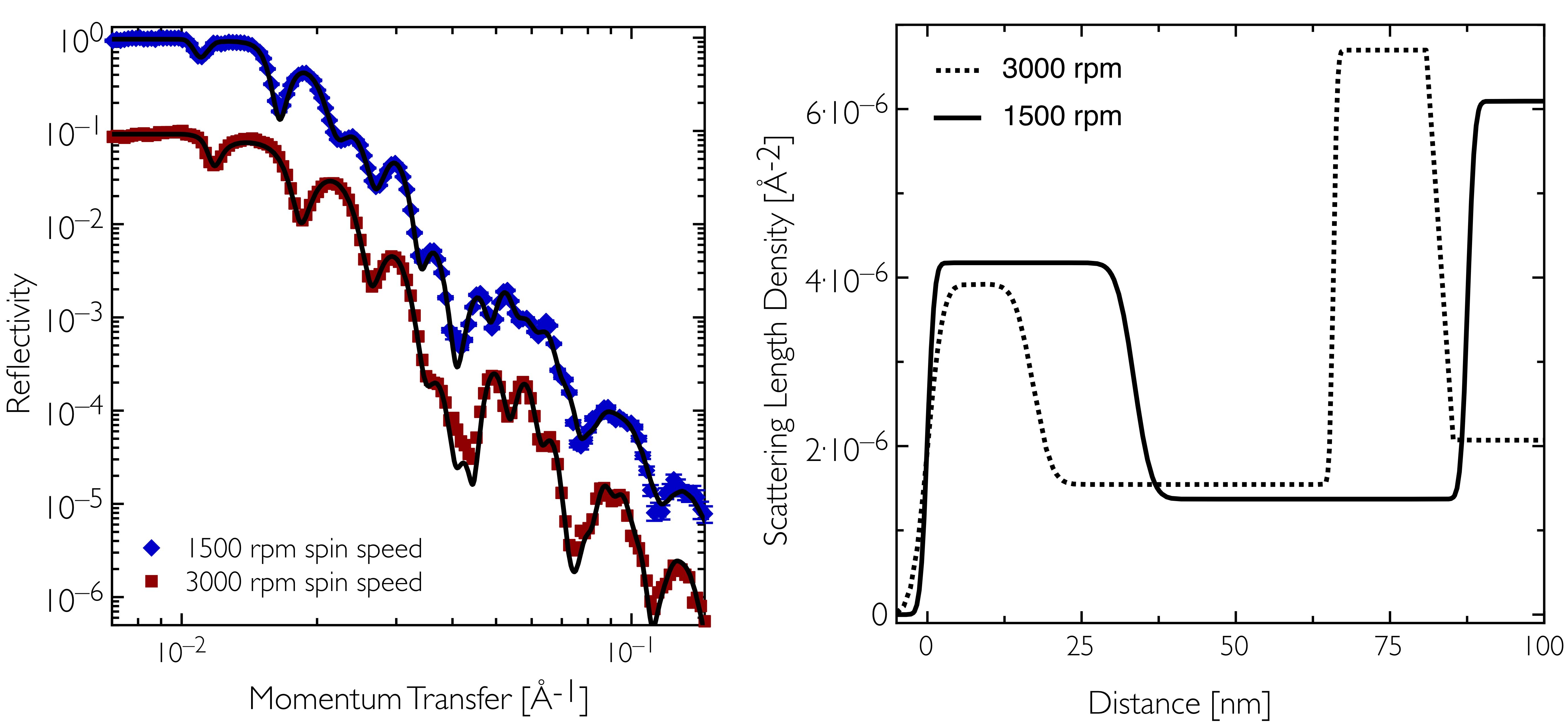


**Figure SI 3.** Neutron reflectivity data for spin coated 1:4 blend layers of PCDTBT:PC_61_BM at spin speeds of 1500 rpm and 3000 rpm. The two reflectivity datasets have been offset by a decade for clarity.


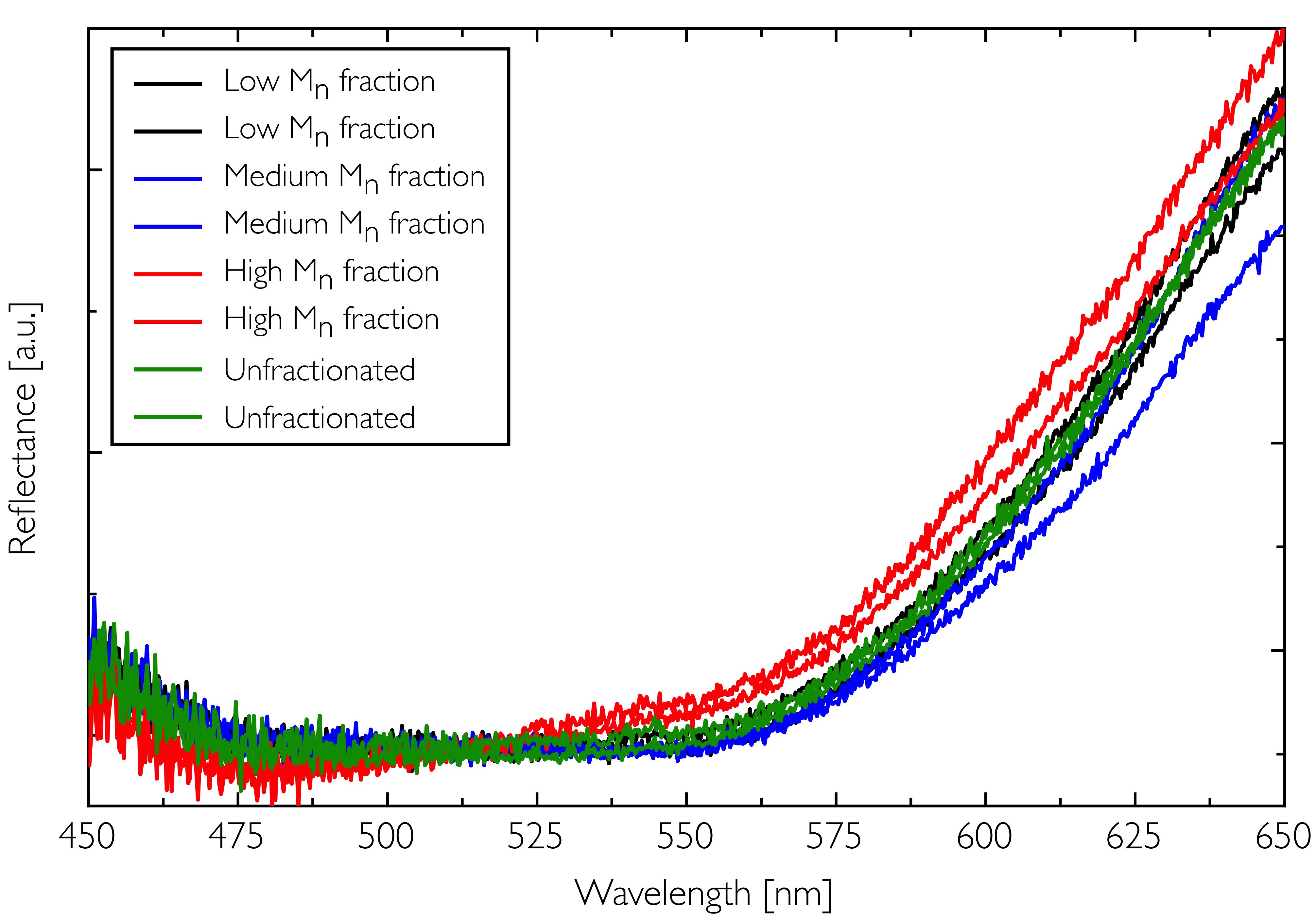


**Figure SI 4.** Reflectance of the OPV devices normalised at 510 nm


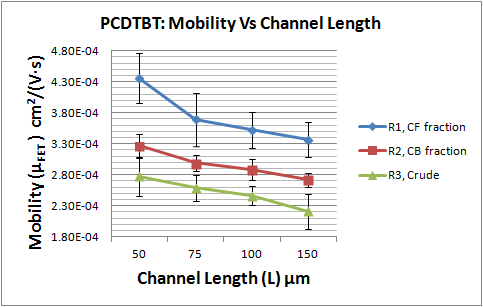


**Figure SI 5.** OFET mobility for the Chloroform and Chlorobenzene fraction for different channel lengths. Average of 15 OFETs with standard deviation shown.

Low Mn (15.0 kDa) Medium Mn (25.1 kDa)


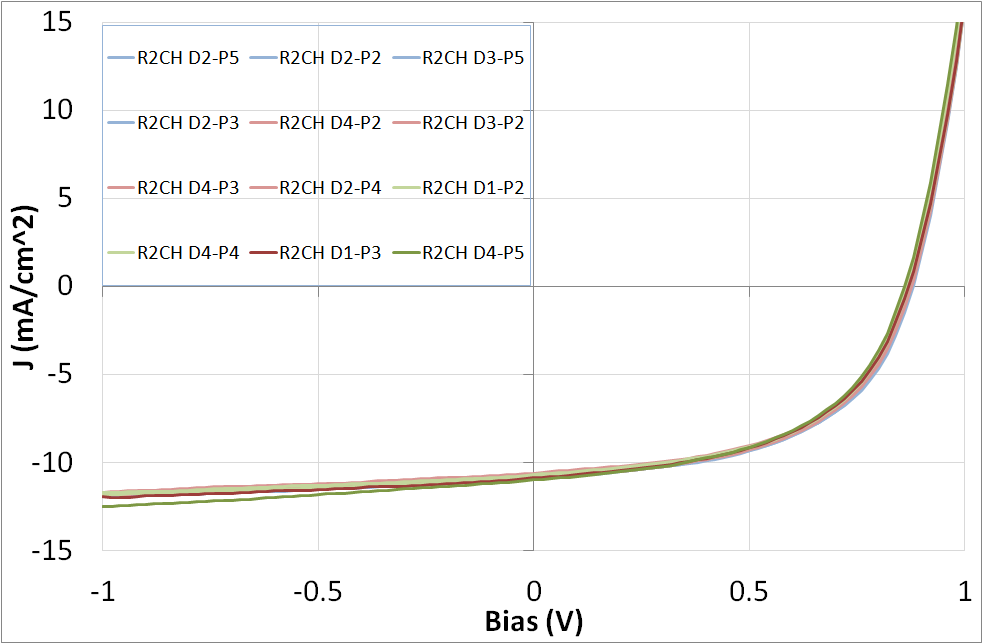

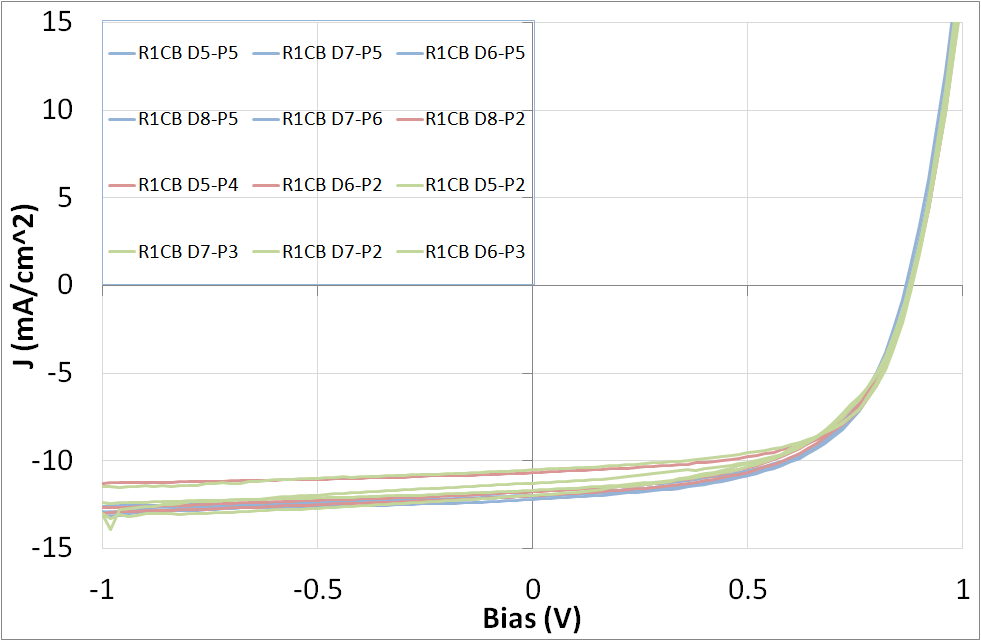


High Mw (31.6 kDa) Unfractionated (27.3 kDa)


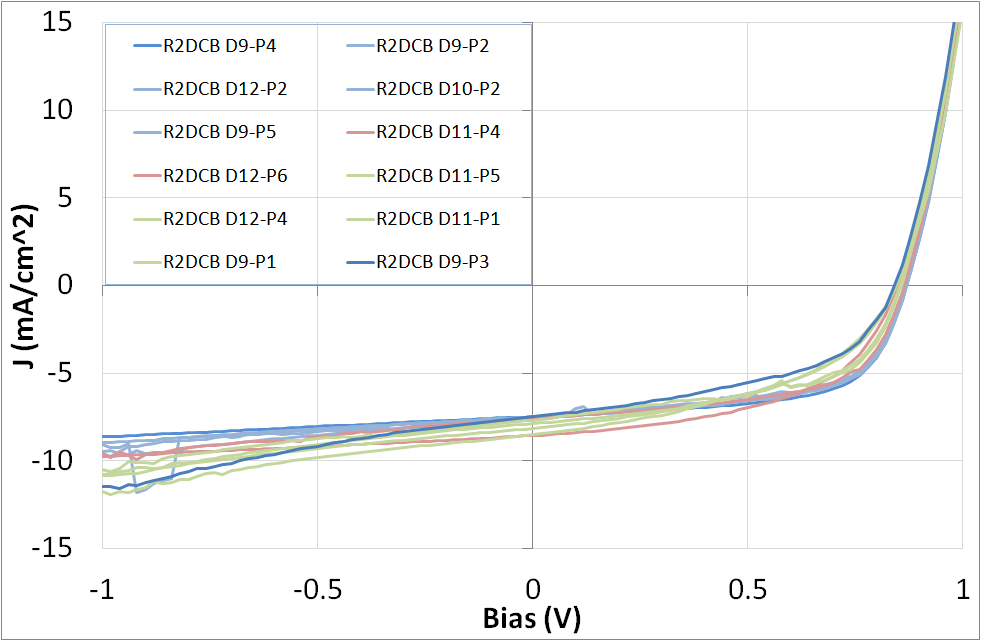

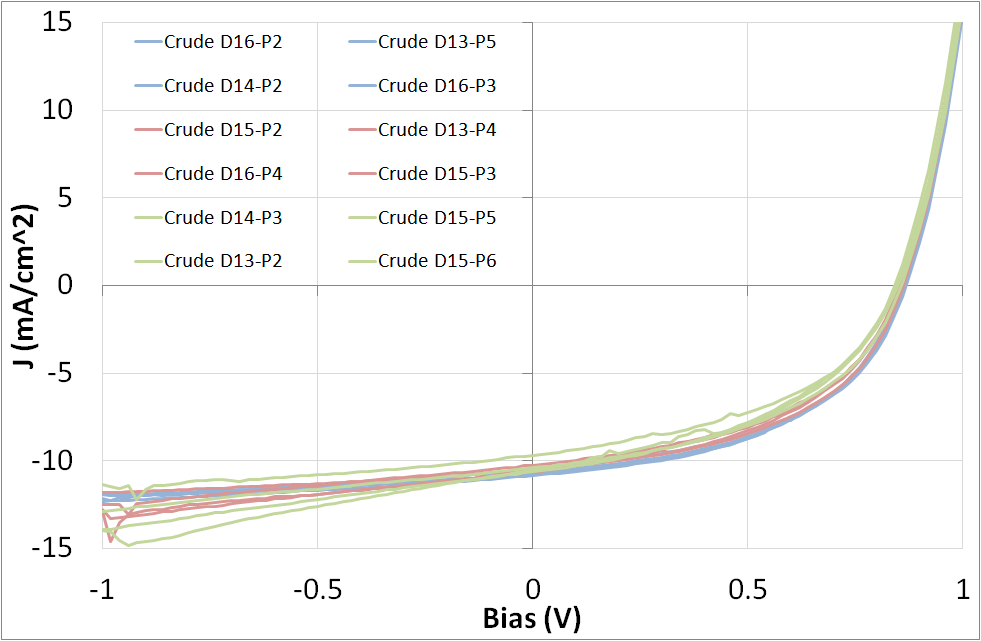


**Figure SI 6.** J-V curves for all devices/pixels used within this analysis for the four different molecular weights of PCDTBT used in the blend devices.


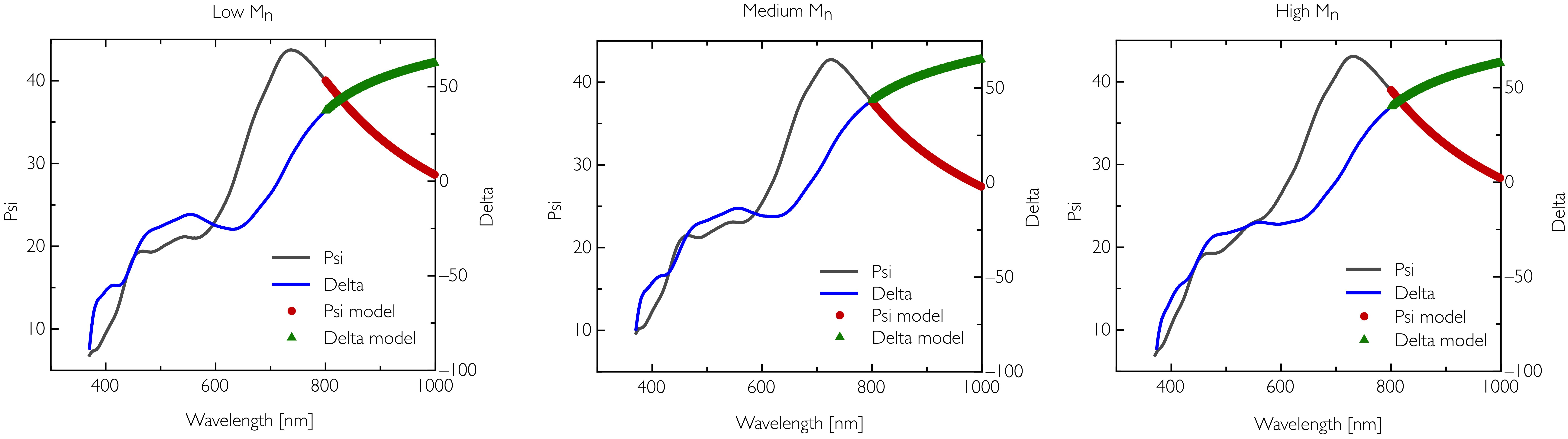


**Figure SI 7.** Ellipsometry data for the fractionated OPV PCDTBT:PC_71_BM blend films with a Cauchy model fitted over the optically transparent region 800 nm -1000 nm; low M_n_  PEDOT:PSS layer 27.4 nm and blend layer 69.6 nm, medium M_n_  PEDOT:PSS layer 27.6 nm and blend layer 65.3 nm, high M_n_  PEDOT:PSS layer 28.0 nm and blend layer 66.8 nm.


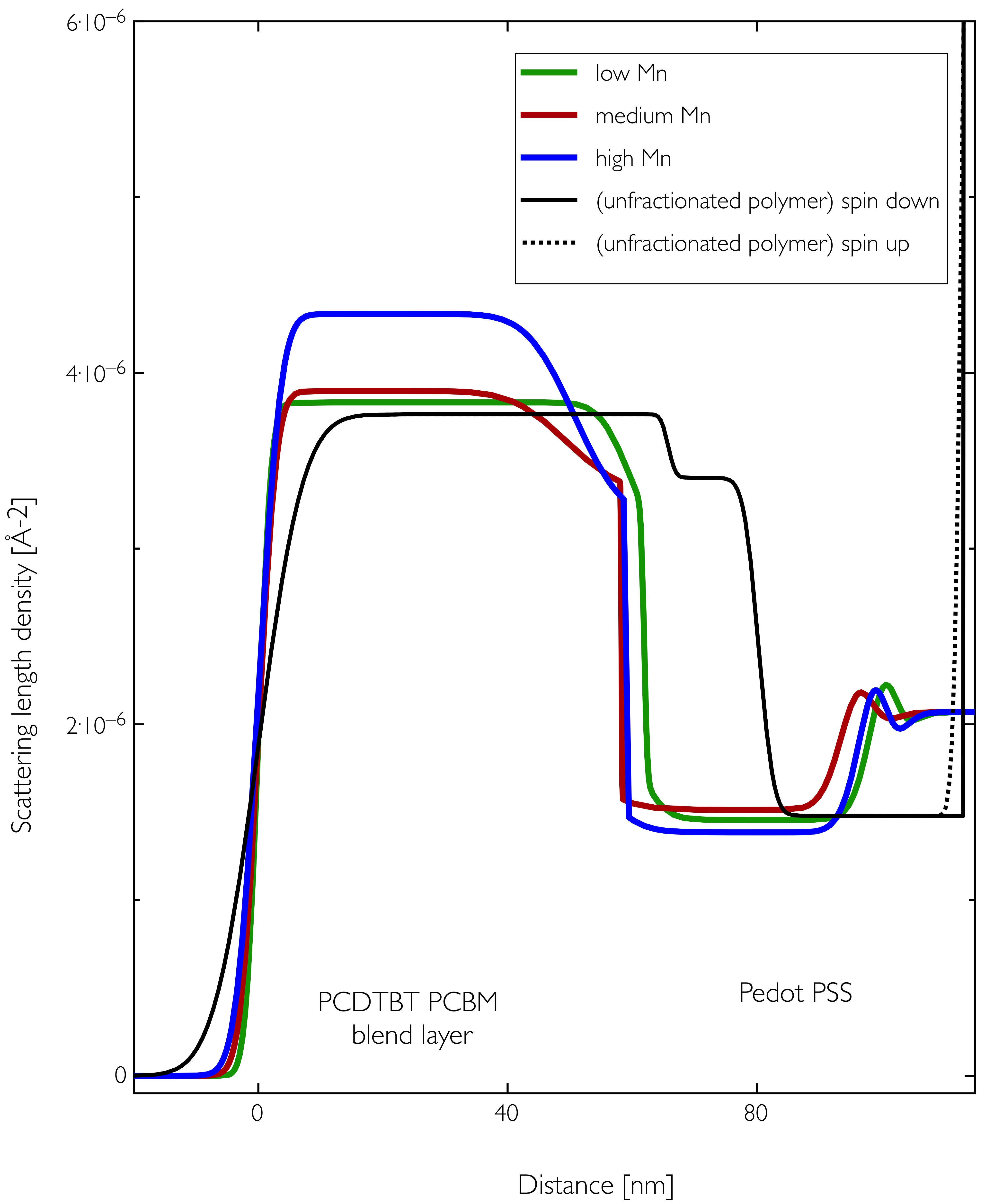


**Figure SI 8.** Neutron reflectivity profiles for spin coated 1:4 blend layers of PCDTBT:PC_70_BM without normalisation to the onset of the PEDOT:PSS layer.


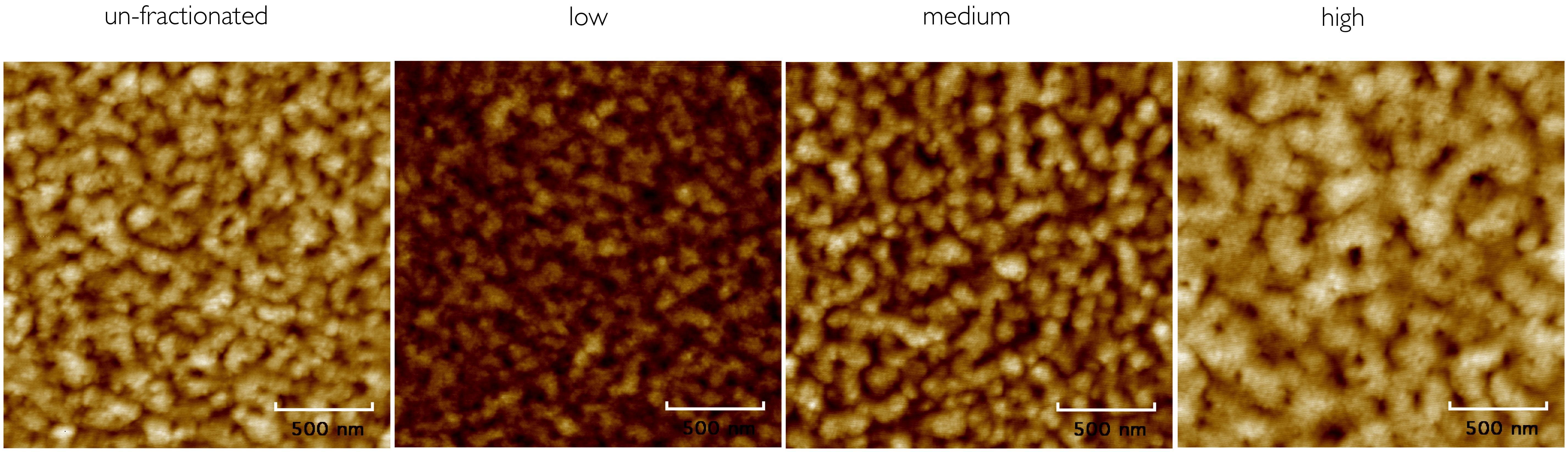
 **Figure SI 9.** Tapping mode atomic force microscopy images of the four samples measured using neutron reflectivity. The RMS surface roughness values are 1.3 nm unfractionated, 1.0 nm low M_w_, 1.2 nm medium M_w_, 1.4 nm low M_w_.
